# Genetic mapping and survey of powdery mildew resistance in the wild Central Asian ancestor of cultivated grapevines in Central Asia

**DOI:** 10.1038/s41438-020-0335-z

**Published:** 2020-07-01

**Authors:** Summaira Riaz, Cristina M. Menéndez, Alan Tenscher, Daniel Pap, M. Andrew Walker

**Affiliations:** 1 Department of Viticulture and Enology, University of California, Davis, CA 95616 USA; 2 Instituto de Ciencias de la Vid y del Vino (ICVV), Universidad de La Rioja-CSIC-Gobierno de La Rioja, Carretera de Burgos Km, 6, Finca La Grajera, Logroño, La Rioja 26007 Spain

**Keywords:** Genetic markers, Plant breeding

## Abstract

Cultivated grapevines (*Vitis vinifera*) lack resistance to powdery mildew (PM) with few exceptions. Resistance to this pathogen within *V. vinifera* has been reported in earlier studies and identified as the *Ren1* locus in two Central Asian table grape accessions. Other PM-resistant cultivated varieties and accessions of the wild ancestor *V. vinifera* subsp. *sylvestris* were soon identified raising questions regarding the origin of the resistance. In this study, F1 breeding populations were developed with a PM susceptible *V. vinifera* subsp. *vinifera* breeding line and a PM-resistant subsp. *sylvestris* accession. Genotyping was carried out with five *Ren1* locus linked SSR markers. A PM resistance locus explaining up to 96% of the phenotypic variation was identified in the same genomic position, where the *Ren1* locus was previously reported. New SSR marker alleles linked with the resistance locus were identified. We report results of PM resistance in multiple accessions of subsp. *sylvestris* collected as seed lots or cuttings from five countries in the Caucasus and Central Asia. A total of 20 females from 11 seed lots and 19 males from nine seed lots collected from Georgia, Armenia, and Azerbaijan were resistant to PM. Three male and one female plant collected as cuttings from Afghanistan and Iran were also resistant to PM. Allelic analysis of markers linked with the *Ren1* locus in conjunction with disease evaluation data found a high diversity of allelic haplotypes, which are only possible via recombination events occurring over a long time period. Sequence analysis of two alleles of the SSR marker that cosegregates with the resistance found SNPs that were present in the wild progenitor and in cultivated forms. Variable levels of PM resistance among the tested accessions were also observed. These lines of evidence suggest that the powdery mildew fungus may have been present in Asia for a longer time than currently thought, giving the wild progenitor *V. vinifera* subsp. *sylvestris* time to coevolve with and develop resistance to this pathogen.

## Introduction

Grapes are the most economically important perennial horticultural crop in the world^1^. The major centers of grapevine diversity are East Asia and North America with up to 30 and 28 reported species, respectively^2,3^. Most cultivated grapes belong to the species *Vitis vinifera*, which is further subdivided into two subspecies subsp. *vinifera* (cultivated grapes) and subsp. *sylvestris* (wild progenitor of cultivated grapes)^4,5^. Molecular evidence indicates that the Caucasus and Northern Iranian region was most likely the first center of domestication^1,6,7^. There is evidence of human presence for more than 20,000 years in the mountains of the southern Caucasus^8^, and transitional types of grapes that include wild forms of the subsp. *sylvestris*, feral and cultivated landraces, and ancient local varieties are common in this region. Grapevines were disseminated from the primary center of domestication to Europe and North Africa following trade routes and reached Central Europe by the first millennium BCE. Cultivated grapes were introduced to China and Japan near the second century BCE^9^. Almost all cultivated *V. vinifera* grapevines are susceptible to powdery mildew with few exceptions^10,11^.

Grape powdery mildew, a fungal disease caused by the obligate biotrophic pathogen *Erysiphe necator*, affects vineyards worldwide. The presence of powdery mildew (PM) resistance in many North American grape species led to the assumption that this pathogen originated in North America and coevolved with endemic grape species. Historical records indicate that PM was introduced to Europe during a period of frequent trade with North America, which included the exchange of plant material^12^. There is no mention of the mildew diseases in Europe prior to the early 1800s. By the mid-19th century, PM was reported throughout Europe and the Mediterranean region on cultivated European grapevine, *V. vinifera* subsp. *vinifera*, which is highly susceptible to this pathogen^13^. Historical records for grape growing regions in the Caucasus and Central Asia are limited to the early 1900s when the renowned Russian geneticist Nikolai Vavilov initiated expeditions to collect germplasm from this region and many others^6,14–16^. However, there is no indication of PM in the Caucasus or Central Asia. Therefore, it was very surprising to find resistance to PM in the wild germplasm from Asia^17^. To date, powdery mildew resistance loci (*Ren4*, *Ren6*, and *Ren7*) have been genetically mapped in two Chinese grape species^18–20^, and multiple accessions from *V. vinifera* subsp. *vinifera*, presumably collected from Central Asia carrying the resistance locus, *Ren1*^11,21^, have been identified. The *Ren1* and *Ren7* loci provide partial resistance with limited hyphal growth and sporulation; the *Ren4* and *Ren6* loci provide complete resistance by limiting the establishment of the fungus via programmed cell death of the invaded cells^10,11,18,20^. The presence of PM resistance in Chinese grape species and *vinifera* cultivars is currently unexplained.

The identification of the grape PM resistance locus *Ren1* in 10 accessions of *V. vinifera* from Central Asia was of great interest to grape breeders and evolutionary biologists. This discovery prompted debate regarding the origins of PM resistance in cultivated grapes, which are typically highly susceptible to powdery mildew, as well as in other Chinese grape species^18,20^. The pathogen defense system of plants is central to the constant battle between hosts and pests and there are multiple means by which plants develop pathogen defense systems. Host plants could gain immunity by coevolving with a given pathogen over a long period of time and undergo direct or indirect selection by humans in the process of domestication. Host plants could also generate variation for resistance against a pathogen through hybridization and subsequent introgression of resistance genes (R-genes) or alleles from a related species to gain immunity; a process referred to as adaptive introgression^22^. Sharing of favorable resistance loci and/or alleles between closely related species via gene flow is also possible if both species encounter similar or related pathogens^23^. There are many examples of plant genes that show signs of adaptive introgression, (reviewed in Vekemans^24^) including genes conferring herbivore resistance in *Helianthus*^25^, and resistance to rust fungus in grasses^26^. Gene flow, from the same or different species, recombination and recombination-like events, and mutations are a few of the key fundamental forces that create novel genotypes by shuffling genetic material and facilitating selection and evolution for a particular trait^27–29^.

One of the significant findings of Riaz et al.^11^ was the identification of PM resistance in two accessions of subsp*. sylvestris*—DVIT3351.27 (collected as seed), and O34-16 (collected as cuttings) from Armenia and Iran, respectively. Both had varying levels of resistance to the disease and carried the allele of the simple sequence repeat (SSR) marker that cosegregates with the *Ren1* locus reported by Coleman et al.^21^. The sequence comparison of the SSR marker allele among different accessions also showed sequence homology; however, the seedling accession DVIT3351.27 did not share alleles with other markers linked to the *Ren1* locus, and it was hypothesized that the genetic origin of its PM resistance might be different than the *Ren1* locus^11^. Without additional linkage mapping work, the sequence similarity between the allele and marker that cosegregates with resistance was considered insufficient evidence to verify whether resistance in the subsp. *sylvestris* seedling accession is homologous to the *Ren1* locus.

One of the key objectives of this study was to confirm that PM resistance in the subsp. *sylvestris* seedling DVIT3351.27 is similar to the *Ren1* locus reported in earlier studies^10,21^, and that it is located in the same genetic position on chromosome 13. The F1 breeding populations were developed by crossing a susceptible *V. vinifera* subsp. *vinifera* female selection 08326–61 with the PM-resistant DVIT3351.27 identified in the earlier study. Results confirmed the resistance locus in DVIT3351.27 is on chromosome 13 where the *Ren1* locus has been reported and new alleles were identified with the linked SSR markers. Identification of new alleles of SSR markers linked to PM resistance prompted us to expand our search to identify additional PM-resistant subsp. *sylvestris* accessions collected from Armenia, Georgia, Azerbaijan, Iran, and Afghanistan. In this study, we report the discovery of PM resistance in multiple accessions of subsp. *sylvestris* that were collected as seed lots or cuttings from five countries. Allelic analysis of markers linked with the *Ren1* locus in conjunction with disease evaluation data offered an opportunity to gain insight into the history of PM resistance in the wild progenitor of cultivated grapes.

## Results

### Disease evaluations

**Fig. 1:**
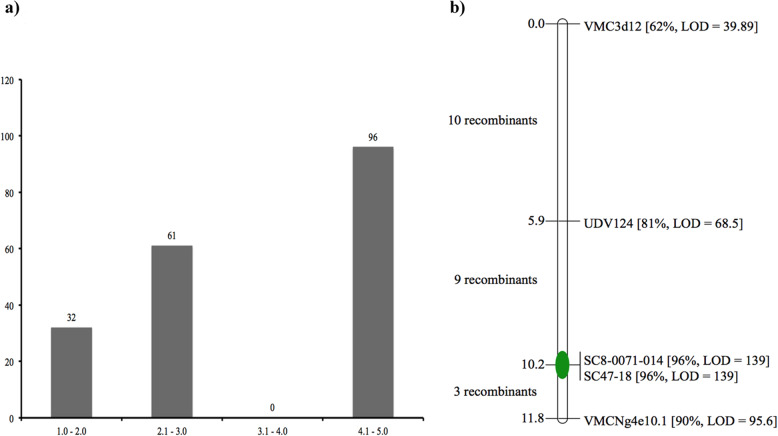
Distribution of disease evaluations and genetic map of accession DVIT3351.27. **a** Distribution of the powdery mildew disease evaluation for 189 seedlings from the three F1 populations. **b** Local genetic map of accession ‘DVIT3351.27’with five markers from chromosome 13. The QTL analysis identified major locus that explains up to 96% variation at marker position SC8–0071-14/SC47–18 with LOD threshold of 139

Two sets of germplasm were evaluated for PM resistance. First set consisted of 189 seedlings from F1 breeding populations. Four biological replicates of 189 seedlings were evaluated in an unsprayed greenhouse with the C-isolate^30^ of *E. necator*. Leaf symptoms were recorded after inspections of the whole plant. Supplementary Table 1 provides the details on the number of seedlings tested, minimum and maximum scores, means, and variances. The susceptible controls ‘Carignan’ and 08326–61 had PM ratings of 4.9–5.0, respectively, in comparison to the PM-resistant parent DVIT3351.27, which had an average score of 2.0. Figure 1a shows the distribution of the phenotypic classes within 189 seedling plants. A total of 93 plants had phenotypic ratings of 1.0–3.0 indicating that none or only a few restricted patches of PM were observed. A total of 96 plants showed ratings of 4.1–5.0, indicating that active and reproducing PM was observed on many leaves and/or plants were highly infected. None of the breeding population had an intermediate rating between 3 and 4.

**Fig. 2:**
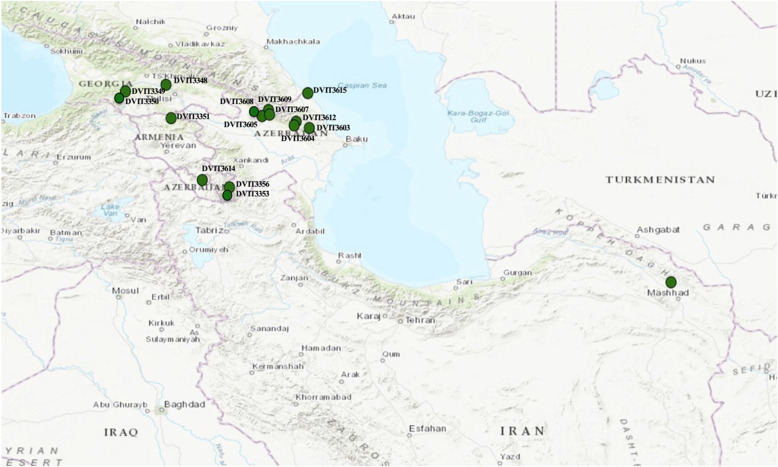
Collection information for the germplasm used in this study. Visual presentation of the collection locations of seed lots and cuttings from Georgia, Armenia, Azerbaijan, and Iran using the GPS coordinates provided in Table 1

The second set of screened germplasm consisted of 125 plants (108 seedlings from 15 seed lots and 17 accessions collected as cuttings) from subsp. *sylvestris* (including DVIT3351.27 and O34-16 identified in the previous study). This set also included five accessions from subsp. *vinifera* that carry the *Ren1* locus (Table 1). The in vitro detached leaf assay using greenhouse grown plants and the C-isolate was used to screen four replicates of each accession, which were examined 14 days after inoculation. Supplementary Table 2 provides details of disease evaluation results. Slightly higher ratings were observed for the detached leaf assay when common reference accessions were tested with both systems (Supplementary Tables 1 and 2). These ratings were used to set a threshold of 3.5 to define a test plant as resistant to PM. There were eight seedlings from different seed lots and one accession collected as cutting with ratings from 3.5–3.88; five of them had a variance of 0.4 among the replicates (Supplementary Table 2). The accession O34-16, which was the subject of two previous studies, was among them. O34-16 was evaluated for PM resistance in the field^11^, and via detached leaf assay and was scored at day 5 after inoculations^31^. Variable results were observed, but overall it had a rating less than 3.5 in earlier screens. Disease evaluation results were not available for three accessions (Sochal, O34-55, and DVIT3609.13) as their leaf quality deteriorated and reliable estimates of symptom expression could not be made. Although Sochal was not tested in this study, it was called resistant based on results of previous studies (Supplementary Table 2). A total of 41 accessions (36 seedlings from different seed lots and five accessions collected as cuttings) had ratings of less than 3.5 and were recorded as resistant. Eight other tested accessions showed high variability among replicates due to different factors and disease severity scores ranged from 3.51–3.88. If we consider them as resistant, then a total of 49 resistant accessions were identified in this study. Six of these were collected as cuttings from Afghanistan and Iran, in 1948 by Dr. Harold P Olmo^11^, and 43 seedlings from 14 seed lots were collected from Armenia, Azerbaijan, and Georgia (Fig. 2).

**Table:** Table 1 List of cultivated and wild accessions of *Vitis vinifera* L. based on geographic origin and collection site

Source countries	Species	Collection location	Seed lot ID	GPS coordinates	Number of unique accessions
Afghanistan, Turkmenistan, and USSR	*V. vinifera*				5
Afghanistan	*V. sylvestris*	Unknown			2
Iran	*V. sylvestris*	Mashad, Razavi Khoransan		36.72587, 59.38915	15
Georgia	*V. sylvestris*	Mukhrani, Georgia	DVIT3348	41.98197, 44.52224	2
		S-8, Georgia	DVIT3349	41.80848, 43.33115	1
		S-8, Georgia	DVIT3350	41.80848, 43.33115	10
Armenia	*V. sylvestris*	Alaverdi	DVIT3351	41.09557, 44.67557	12
		H-45	DVIT3353	39.25591, 46.39120	4
		H-45	DVIT3356	39.25591, 46.39120	1
		Nabran	DVIT3615	41.76033, 48.69828	10
Azerbaijan	*V. sylvestris*	Bassal, Ismayilli Dist	DVIT3603	40.83306, 48.74111	12
		Bassal, Ismayilli Dist	DVIT3604	41.00028, 48.35583	4
		Kungut Post, Sheki	DVIT3605	41.31194, 47.54250	8
		Kungut Post, Sheki	DVIT3607	41.31194, 47.54250	9
		Bash Keldek, Sheki	DVIT3608	41.15278, 47.34972	6
		Bayan, Oguz	DVIT3609	41.19306, 47.57083	8
		Gurubanfendi, Ismayilli	DVIT3612	40.90194, 48.29694	14
		Ayridj, Shahbuz	DVIT3614	39.44611, 45.59194	7
Total					130

Samples from Georgia, Armenia, and Azerbaijan were collected as seed lots during two collection trips. Each seed lot was given an identification code linking it to GPS coordinates for future reference. The samples from Iran and Afghanistan were collected as cuttings during a trip made by Dr. Harold P Olmo in 1948. No location information is available for samples from Afghanistan. Five cultivated accessions were either collected and/or acquired at different time intervals from other collections in the world

### Genotyping, local genetic linkage map, and QTL analysis

The 189 seedlings from the F_1_ populations based on DVIT3351.27 were used to develop a localized genetic map using five polymorphic markers (VMCNg4e10.1, Sc47–18, SC8–0071–14, UDV124, and VMC3d12) spanning a genetic distance of ~12 cM on chromosome 13. These five markers were linked to the *Ren1* locus in previous studies^10,11^. Supplementary Table 3 shows the genotypic profile of all F_1_ seedlings. As described in the Methods, genotypic analysis in earlier studies was carried out with a different fragment analyzer and size standards for allele calls; therefore, we expected the allele calls to be slightly different from the previous reports. There was a 4-base pair (bp) downward shift in allele calls for marker UDV124, 3 bp for VMCNg4e10.1 and Sc47–18, and 2 bp for marker SC8–0071–14 and VMC3d12. Our expectation was that with the cosegregating marker SC8–0071–14, the allele 141 would be linked to resistance in the F_1_ population, as we have observed in other accessions that carry the *Ren1* locus. However, a quick scan of the genotypic and phenotypic data revealed that it was allele 143 cosegregating with marker SC8–0071–14. A set of different alleles with other *Ren1*-linked markers was also identified in the F_1_ populations.

The total length of the genetic interval based on five resistance-linked markers in the resistant parent DVIT3351.27 was 11.8 cM. No recombination was observed between markers Sc47–18 and SC8–007–14, three recombinants were identified between Sc47–18 and VMCNg4e10.1, and there were nine recombinants between SC8–007-14 and UDV124 (Fig. 1b). The interval mapping (IM) algorithm was used to carry out QTL analysis. A significant QTL that explained up to 96% of the variation (LOD = 139) was identified that peaked at markers Sc47–18 and SC8–007-14 (Fig. 1b). This clear result showed that the subsp. *sylvestris* accession DVIT3351.27 has PM resistance at the same genetic position that the *Ren1* locus from ‘Kishmish vatkana’^10^ was mapped to.

Upon discovery of new alleles for the five SSR markers in phase with the *Ren1* resistance allele in the accession DVIT3351.27, we expanded the search and genotyped 123 accessions that were collected as seeds or cuttings during three different collection trips from 1948 to 2010 (Table 1). The same set of five markers was used and alleles were assigned. Supplementary Table 4 shows the genotyping results of 123 new and seven previously reported accessions that were used as references. Table 2 provides the summary of different allelic categories and the number of resistant and susceptible accessions within each category. Both previously reported and newly discovered SSR marker alleles were observed in subsp. *sylvestris* accessions collected from different geographic locations. Both resistant and susceptible plants were identified within groups that carried either allele 141, or 143, or both alleles together (as was the case for DVIT3351.27, which was used to map the locus), and/or 143/143. A total of 52 plants were susceptible and did not carry either allele of SSR marker SC8–0071-14; however, they had resistance-linked alleles with other markers. There was only one accession, that was resistant but did not have resistance-linked alleles with the five tested markers. With the exception of one other seedling, DVIT3351.37, no other seedling line showed a complete stretch of the 11.8 cM haplotype that was similar to DVIT3351.27. None of the subsp. *sylvestris* seedling lines carried the haplotype of linked alleles that were observed in subsp. *vinifera* accessions. However, many subsp. *sylvestris* seedling lines had subsp. *vinifera* alleles for one to three markers (S. Table 4). This observation shows that frequent recombinations in obligatory outcrossing populations of subsp. *sylvestris* break apart the haplotypes over generations and cause a rapid decay of linkage disequilibrium. On the other hand, results for seven accessions of subsp. *vinifera* identified in a previous study^11^, show that the introgressed haplotype was intact and maintained owing to possible clonal propagation of superior selections by farmers over centuries.

**Table 2:** Detail of resistant and susceptible accessions that carried allele 141 and/or 143 with SSR marker SC8–0071-14 that cosegregates with the powdery mildew resistance *Ren1* locus

Allelic categories	Resistant	Susceptible	Total
Accessions with allele 141	10	9	19
Accessions with allele 143	28	9	37
Accessions with allele 141/143	4	1	5
Accessions with allele 143/143	8	1	9
Accessions without allele 141 or 143	1	52	53
Total	51	72	123

To verify the wild status of germplasm tested in this study, the flower phenotype was recorded from plants growing in the field (Supplementary Table 2). Wild subsp. *sylvestris* is dioecious with male and female plants only. From a total of 108 seedling plants (including DVIT3351.27) from 15 seed lots, 50 plants were female, 47 were male, seven were hermaphrodite, and four did not flower. A hermaphrodite flower phenotype was observed in plants from two seed lots (DVIT3348 and DVIT3350) collected from Georgia in 2007; one hermaphroditic seedling DVIT3350.13 was resistant to PM. Over all, 20 PM-resistant females (11 seed lots) and 19 males (nine seed lots) were identified. Among the plant material collected as cuttings from Iran and Afghanistan in 1948, three PM-resistant plants were male, one female and three were hermaphroditic.

### Genetic diversity measures

Five SSR markers were polymorphic for the set of 130 plants and 17–21 alleles were observed (Table 3). The observed heterozygosity was slightly lower than the expected heterozygosity for four markers and higher for marker VMC3d12. Even though we observed increased levels of genetic variation for five linked markers, there was bias for a higher frequency of a few select alleles for each marker. Four alleles for marker VMC3d12 had a frequency of higher than 5 and cumulatively they contributed to 70% of the allele frequency. There were eight alleles for marker SC8–0071-14 and UDV124 that contributed 86% and 75% of the frequency, respectively. For marker VMCNg4e10.1 and Sc47–18, four and five alleles had a frequency of 83% and 75%, respectively.

**Table 3:** Genetic diversity estimates of 130 accessions with five markers that are in linkage with the powdery mildew resistance *Ren1* locus

Marker	Na	H_O_	H_E_	Allele size (bp)	Allele frequency	Absolute count
VMCNg4e10.1	17	0.74	0.80	186	2.71	7
				233	0.39	1
				236	4.26	11
				245	0.78	2
				248	36.82	95
				251	1.16	3
				254	1.55	4
				**257**	**5.43**	**14**
				**260**	**19.38**	**50**
				263	12.79	33
				269	8.91	23
				275	2.33	6
				278	0.39	1
				281	0.78	2
				283	0.39	1
				314	0.39	1
				320	1.55	4
Sc47–18	17	0.81	0.86	200	2.73	7
				204	8.98	23
				213	5.08	13
				214	13.28	34
				215	3.13	8
				217	0.39	1
				220	2.73	7
				224	0.39	1
				228	0.39	1
				233	12.11	31
				234	7.42	19
				237	1.56	4
				238	6.25	16
				**239**	**29.69**	**76**
				242	1.17	3
				**246**	**4.30**	**11**
				248	0.39	1
SC8–0071-014	18	0.85	0.89	**141**	**9.23**	**24**
				**143**	**23.08**	**60**
				155	1.54	4
				157	5.77	15
				159	3.08	8
				160	1.15	3
				161	3.46	9
				163	11.54	30
				165	0.38	1
				167	9.62	25
				170	0.77	2
				171	8.46	22
				173	0.38	1
				177	0.38	1
				191	0.38	1
				197	2.69	7
				199	6.92	18
				203	11.15	29
UDV124	21	0.85	0.91	183	8.91	23
				185	20.16	52
				187	0.39	1
				189	2.33	6
				191	2.71	7
				193	11.24	29
				194	1.55	4
				195	0.78	2
				201	5.81	15
				202	0.39	1
				203	4.65	12
				**208**	**3.49**	**9**
				210	5.04	13
				**212**	**10.08**	**26**
				214	7.36	19
				216	6.59	17
				218	1.55	4
				225	1.16	3
				227	2.71	7
				229	2.71	7
				275	0.39	1
VMC3d12	19	0.87	0.84	192	16.54	43
				194	5.00	13
				**196**	**3.08**	**8**
				197	1.92	5
				198	29.62	77
				199	1.92	5
				200	0.77	2
				**201**	**19.23**	**50**
				202	3.46	9
				204	2.69	7
				209	0.38	1
				210	4.23	11
				215	4.23	11
				216	1.54	4
				219	0.77	2
				220	0.77	2
				221	0.77	2
				225	1.92	5
				235	1.15	3

‘Na’ is No. of alleles per locus, ‘HO’ is observed heterozygosity, and ‘HE’ is expected heterozygosity. Allele frequencies and absolute count for each allele were also calculated. The alleles in bold are linked with the resistance locus

The PCoA analysis detected three clusters (Supplementary Fig. 1). Group ‘a’ consisted of 20 samples, mostly from Armenia, Iran, and Afghanistan and included five subsp. *vinifera* accessions. All of them had allele 141 for marker SC8–0071-14 and displayed both resistant and susceptible phenotypes. Group ‘b’ consisted of 60 samples that displayed diverse allelic composition with marker SC8–0071-14. Both alleles (141 and/or 143) showed up with resistant and susceptible phenotypes; homozygous lines with 143/143 alleles had both resistant and susceptible phenotypes, and resistant lines that carried both 141/143 alleles. Most samples in this group came from different seed lots collected from three countries. Similarly, group ‘c’ consisted of 50 samples that were collected as seeds from Armenia, Azerbaijan, and Georgia, and carried allele 141 or 143 and both resistant and susceptible phenotypes were associated with those alleles.

### Sequencing of the resistance-linked alleles that cosegregate with the SC8–0071-014 SSR marker

**Fig. 3:**
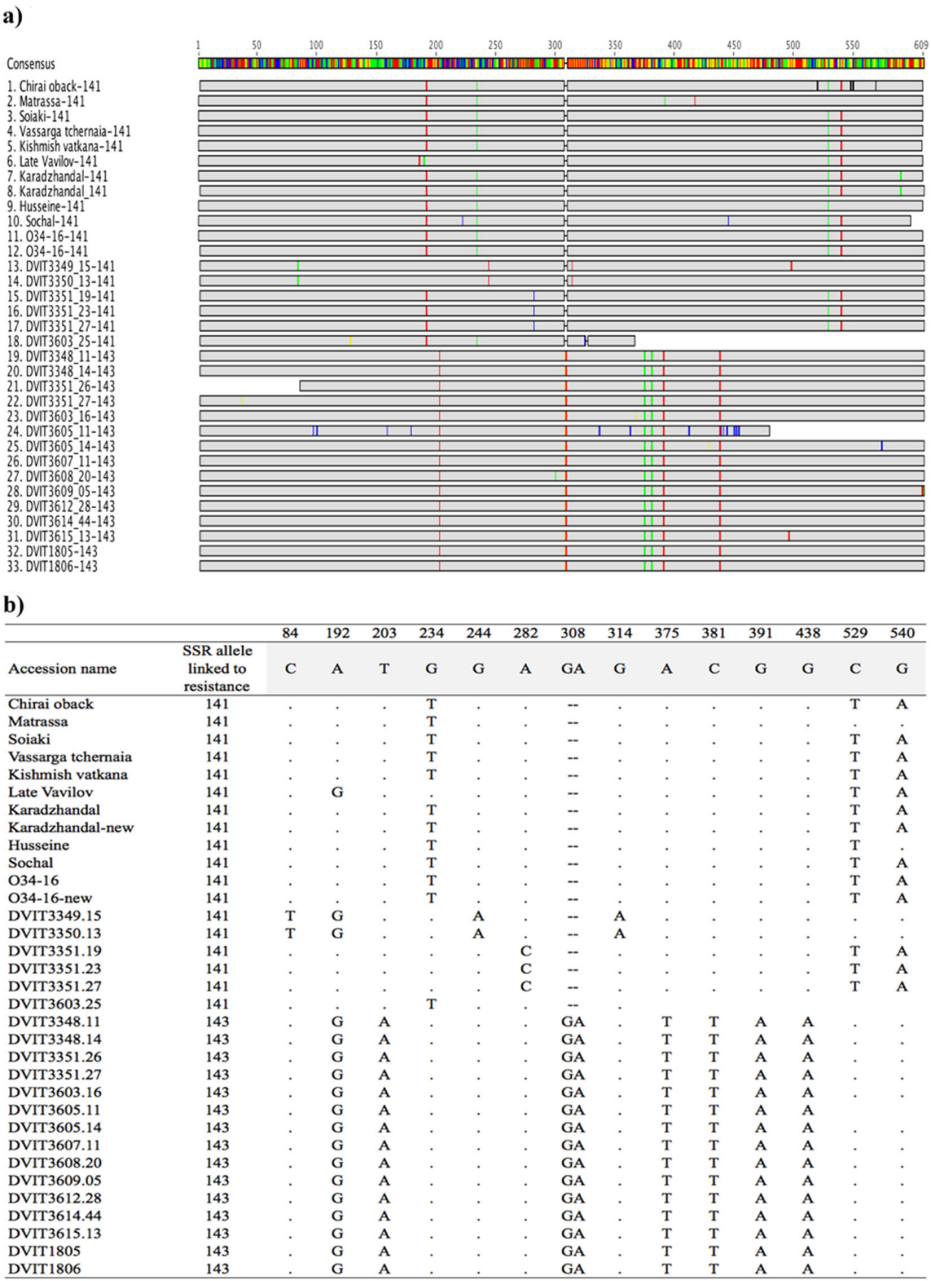
Sequence comparisons of the alleles of SSR marker SC8-0071-014. **a** Sequence comparison of a 610(bp) region associated with the 141 or 143 allele of SSR marker SC8–0071-014 that cosegregates with the *Ren1* locus. Samples 1–18 have allele 141, and 19–33 have allele 143. Sequences of samples 1–12 were published in an earlier study (Riaz et al. 2013a) with the exception of sample 8 and 12 (Karadzhandal and O34-16), which were repeated in this study as a control to compare with the previous work. First 10 samples are *V. vinifera*. subsp. *vinifera* and 11–33 are *V. vinifera*. subsp. *sylvestris*. **b** Position and type of single nucleotide polymorphism that were identified in the sequences of 33 accessions

A 609 bp region that includes the resistance associated 141 and 143 bp alleles from marker SC8–0071-014 was sequenced for the 20 newly discovered PM-resistant accessions (18 belonged to 12 different seed lots and two accessions were collected as cuttings). The sequences of 10 accessions reported in an earlier study were included as a reference 11. The sequencing of allele 141 was repeated for two accessions Karadzhandal and O34-16 and their previous sequences were also included as a control. The seedling DVIT3351.27 has both allele 141 and 143; both were sequenced and included in the study. Figure 3a, b shows the alignment of 33 sequences and map out positions of single nucleotide polymorphisms (SNPs) among the sequences.

There was no discrepancy between the new and old sequences of the two accessions, Karadzhandal and O34-16. The comparison of sequences among different plants found that four SNPs at position 192 (G - > A), 234 (G - > T), 529 (C - > T), and 540 (G - > A) were associated with allele 141. A total of seven accessions (six subsp. *vinifera* and one subsp. *sylvestris*) carried these four SNPs (Fig. 3b). The cultivar Matrassa carried two SNP at position 192 and 234, and Husseine had the SNPs at position 192, 234, and 529. The sequence of allele 141 of seedling DVIT3351.27 and two other seedlings from the same seed lot had the SNP at position 282 (A - > C) and also carried SNPs at positions 529 and 540, which were observed in the subsp. *vinifera* accessions. A total of five SNPs were present in 15 plants that had allele 143. The SNP positions 203 (T - > A), 375 (A - > T), 381 (C - > T), 391, and 438 (G - > A) were in complete association with the 2-bp GA indel at position 308.

## Discussion

### Identification of additional powdery mildew resistant subsp. sylvestris accessions

This study builds upon earlier work by Riaz et al.^11^ and sought to discover additional PM-resistant germplasm that shared the alleles of SSR markers linked to the PM resistance locus *Ren1*, identified in earlier work by Hoffmann et al.^10^. Ten new PM-resistant accessions were identified; two of them belonged to the subsp. *sylvestris* (DVIT3351.27 and O34-16) collected from Armenia and Iran^11^. Both subsp. *sylvestris* accessions carried the alleles of SSR markers that cosegregated with the *Ren1* locus. The sequences of the respective alleles were also homologous with the *Ren1*-carrying subsp. *vinifera* accessions. However, questions remain, do these subsp. *sylvestris* accessions have PM resistance because of the *Ren1* locus that was genetically mapped in Kishmish vatkana, a subsp. *vinifera* cultivar? Was resistance acquired after the supposed introduction of PM into the Old World or was PM present in the Central Asia allowing the wild progenitor to evolve disease resistance over an extended period and be domesticated into cultivated forms? In this study, we developed F_1_ breeding populations, carried out disease evaluations and developed a localized genetic map with markers in linkage with the *Ren1* locus on chromosome 13 to verify that PM resistance in subsp. *sylvestris* accessions was homologous to the *Ren1* locus. Results verified that PM resistance in subsp. *sylvestris* is localized to the same genomic region where *Ren1* was mapped in the subsp. *vinifera* accession.

A surprising result was a new set of SSR markers alleles linked to the PM resistance in the male seedling plant DVIT3351.27. Thus, the search was expanded to 107 seedlings from 15 seed lots of subsp. *sylvestris* (collected from Armenia, Azerbaijan and Georgia) and 16 accessions collected as cuttings from Iran and Afghanistan in 1948. A total of 43 seedlings and six accessions with resistance to PM based on a detached leaf assay were identified. These results indicated that PM resistance is prevalent in the Caucasus and neighboring countries in Central Asia, which is considered to be the center of grape domestication^9,32,33^. In Central Asia, both subspecies form a genetic and taxonomic continuum without breeding barriers, which can result in spontaneous hybrid forms where their ranges merge. The general consensus is that subsp. *sylvestris* is dioecious in nature with anemophilous pollination while the domesticated grape is hermaphroditic^4^. In grapes, flower phenotype is controlled by a single major locus with three alleles: male (M) dominant to hermaphrodite (H), which is dominant to the female (F)^34^. The most recent model sheds new light on the evolution of the sex locus and provides evidence that the males and females observed in the wild arose from hermaphroditic ancestors^35^. Gene flow from hermaphroditic cultivated forms would not result in male plants in the progeny and instead a 1:1 ratio of hermaphrodite to female vines is expected. However, gene flow from wild to cultivated forms with a *sylvestris* male pollinating hermaphrodite or female plant would yield 50% male plants in the progeny. One also expects to observe 25% of progeny with hermaphroditic flowers if a hermaphroditic maternal parent was pollinated by *sylvestris*. Gene flow could happen in both directions where ranges of both subspecies overlap. Comprehensive collection notes and understanding the genetics of the flower phenotype helps to identify true *sylvestris*. In this study, seeds of wild *sylvestris* were collected in two trips and special attention was paid to make collections from areas that were away from the commercial grape growing regions with only male and female plants in the area. Field assessments of flower phenotype among 108 accessions from 13 seed lots (including DVIT3351) found only male and female plants in the mix, indicating that most likely these seedling plants represent subsp. *sylvestris* with no potential contamination from the cultivated subsp. *vinifera* (Supplementary Table 2). All seedling plants from seed lot DVIT3351 collected from Armenia were either male or female. Based on the flower phenotype model, we can assume that the maternal parent was not hermaphroditic and other potential pollinators were male plants in that area. The transcriptome data comparison of different PM-resistant accessions in a previous study^31^ also placed seedling DVIT 3351.27 in a phylogenetic clade that was distinct from subsp. *vinifera* cultivars. The accession DVIT 3351.27 was also part of the study that used phased sex haplotypes to define the sex locus of the *Vitis* genus^35^. Based on these results, we could infer that the genetic map of the male seedling (DVIT3351.27) potentially represents the *Ren1* locus in a true *sylvestris*. Only two seed lots were identified with hermaphroditic seedlings in the mix (DVIT3348 and DVIT3350, both collected from Georgia) indicating potential gene flow from the cultivated *vinifera* (Supplementary Table 2). In the 17 accessions that were collected as cuttings from different locations in Iran and Afghanistan, male, female, and hermaphrodite flower phenotypes were observed, indicating that many of them are likely hybrid feral types with influence from cultivated *vinifera* accessions. Not much could be said about the influence of *vinifera* for the six male accessions that were collected as cuttings in absence of detailed collection notes of the mother plant when cuttings were taken in 1948.

Earlier studies have utilized molecular analysis to decipher the genetic diversity within both subspecies, infer their relationship to each other to understand the process of domestication^36,37^, and provide evidence of gene flow between local s*ylvestris* and cultivated accessions^1,7^. The events of domestication that occur across different cultures and geographies are complex and have four phases. Phase one refers to active human stewardship, collecting and harvesting favorable wild germplasm; phase two is purposeful cultivation of the crop; phase three is dispersion to new areas; and phase four is purposeful breeding^38^. In general terms, domestication is associated with the series of morphological changes that can include transition to larger fruits or grains, robust plants, better plant structure, properties of seed dispersal and dormancy, and other important characteristics that fit the norm and culture of a region^39^. The domestication history of perennial crops is further complicated by the fact that perennial crops have long generation times with a separate juvenile phase, thus reducing the number of cycles of reproduction. Many of them are also clonally propagated^40^. Studies of crop domestication often include the evaluation of different evolutionary processes, like genetic effects of bottlenecks and the detection of selection signatures for any particular trait^41,42^.

The germplasm reported on in this study was part of two previous studies that used diversity estimates with unlinked SSR markers from 19 grape chromosomes to gain a better understanding of the domestication process^7,11^. Both reports concluded that there is limited differentiation between the two subgroups of *V. vinifera*. In addition, thus far 10 subsp. *vinifera* accessions that possess the *Ren1* haplotype have been identified^10,11,21^, and based on collection records, these accessions represent divergent geographical areas from the Caucasus and Central Asia. It is reasonable to expect that there may be many more cultivated and wild forms with PM resistance awaiting discovery. Hybridization has the potential to introduce new alleles at multiple unlinked loci and shape a species evolution^43,44^. The PM locus linked alleles of SSR markers reported in this study could be used as a diagnostic tool to identify germplasm that could be used in studies to understand the process of natural and artificial selection during domestication for certain traits like flower sex, berry color, cluster shape and size, and horticultural traits such as adaptation to environmental changes^45^.

### Evidence of shared ancestral variation for PM resistance

The multistep complex process of domestication leaves selection footprints like specific alleles, admixed populations that carry those alleles, evidence of recombination that could lead to weak linkage disequilibrium (LD), and sequence similarity of the genomes^46,47^. As most of the germplasm was collected as seeds, we had the chance to view the past and estimate the level of diversity of male plants around a given female plant, as well as find evidence of recombination. Two alleles (141 bp and 143 bp) of the *Ren1* cosegregating marker were observed in different combinations in both PM-resistant and susceptible seedlings (Table 2). When we compared the results of five linked markers, a total of 45 susceptible plants shared alleles with the resistant haplotype of DVIT3351.27 at one or more markers. None of the subsp. *sylvestris* accessions had the complete haplotype except one full/or half sibling of DVIT3351.27 from the same seed lot. Many subsp. *sylvestris* accessions had alleles with SSR markers that were observed in the subsp. *vinifera* accessions, but none of them carried the complete haplotype of cultivated types (Supplementary Table 4). These observations provide evidence for recombination events (a key force to weaken linkage disequilibrium) that broke down a particular haplotype in natural populations of subsp. *sylvestris*. Wild forms are dioecious with male and female plants thus ensuring that the forces of recombination shuffle genomes in each generation and create new haplotypes. The extent of PM resistance in the Caucasus and Central Asia, and the corresponding allelic data suggest that this resistance is the product of thousands of years of evolution that resulted in existing local *Ren1* haplotypes in subsp. *sylvestris*.

The movement of powdery mildew resistance from wild to cultivated forms is certainly not a result of adaptive introgression from other grape species after domestication. The process of adaptive introgression leaves selection signatures in the recipient genomes that can be identified by comparing whole-genome data^48–50^. In this study, we identified and verified the position of the *Ren1* locus on chromosome 13 in the wild progenitor (subsp. *sylvestris*), in the same genomic location and with the associated markers that were reported in the cultivated grape, Kishmish vatkana (subsp. *vinifera*)^10^. We also identified 43 other PM-resistant accessions that typify true subsp. *sylvestris* (male and female plants), and six most likely wild feral types (hermaphroditic plants) collected from different locations within the center of domestication and display different combinations of alleles of linked markers. Both lines of evidence suggest that wild progenitor subsp. *sylvestri*s may have developed PM resistance over a long time, possibly thousands of years. It is also possible that resistance was introduced to cultivated *vinifera* in certain regions at the time of domestication. Once selected, the *Ren1* haplotype stayed intact in subsp. *vinifera* accessions due to the practice of clonal propagation. Some of resistant lines may have been used in intentional breeding as well. An earlier study by Riaz et al.^11^ also observed an unbroken 26 cM long haplotype with six SSR markers in six subsp. *vinifera* accessions that carried the *Ren1* locus. They also identified a first-degree parent progeny relationship between Vassarga tchernaia and Kishmish vatkana with Thompson seedless as the paternal parent, and other second-degree relationships among resistant accessions that were seeded and seedless^11^. These results indicate intentional breeding efforts in different regions of Asia. However, we do not know if breeding was focused on PM resistance, the history and age of PM-resistant cultivars, and why there appears to be no mention of the disease in historical records from this region.

We also carried out sequence analysis on the two alleles of the SSR marker SC8–0071-14 that cosegregate with the *Ren1* resistance locus. The alignment of the 610 bp sequence from previously published and new accessions reported in this study offered only a small window to evaluate the sequence variations between the two subspecies. Overall the sequences were similar except for a set of three SNPs that were present in many subsp. *vinifera* accessions including subsp. *sylvestris* O34-16 that carried marker allele 141; five SNPs that were present in all of subsp. *sylvestris* accessions that carried allele 143; and one SNP at position 192 was also observed in three accessions with allele 141. The only conclusion that could be made from the sequence comparisons is that there is a lot of sequence homology in two alleles of the marker that cosegregates with the *Ren1* locus. Future studies should focus on developing a large-scale genome wide SNP database to gain better understanding of PM resistance from wild ancestors and cultivated types.

In general, unlinked markers are used to estimate genetic variation by comparing levels of heterozygosity, allele frequencies and other diversity measures to gain insights into the structure and history of populations. In this study we used five linked markers that cover ~12 cM to identify additional accessions with PM resistance. The allelic data of subsp. *sylvestris* populations indicated a relatively low linkage disequilibrium (LD) reflected by the diverse range of haplotypes. The weak LD prompted us to compare the H_O_ and H_E_ and calculate the frequencies of alleles linked to PM resistance. Slightly lower levels of H_O_ than H_E_ in the subsp. *sylvestris* accessions indicated a reduced level of genetic variation, likely due to inbreeding as a result of geographic isolation. A low level of diversity was also evident from the allele frequencies where only a few selected alleles for each marker contributed to 70–86% of allele frequency. Reduced levels of diversity in *sylvestris* have also been noted in other studies^36,37,45^. The subsp. *sylvestris* is considered endangered in many parts of the world due to human population pressure and man-made and natural geographical barriers that lead to isolation and reduced gene flow within and among different groups. The PCoA analysis identified cultivated accessions clustered in three groups: with other accessions possessing allele 141, and with both resistant and susceptible phenotypes. The most divergent group consisted of 60 accessions and contained six different allelic combinations with the 141 and 143 alleles of the SSR marker cosegregating with resistance. It would be of great interest to collect and analyze more germplasm, both cultivated and wild, for future studies.

### Presence of powdery mildew in Central Asia and China

In this study, we identified 39 seedlings and four accessions of *V. vinifera* subsp. *sylvestris*, the progenitor of the cultivated *V. vinifera* subsp. *vinifera*, that possess PM resistance. This germplasm was collected from a wide geographical region indicating that PM resistance is common in the first center of grape domestication. Previous studies also have reported PM resistance in cultivated forms^10,11,21^. Powdery mildew resistance was also identified in two Chinese grape species and genetically mapped on different grape chromosomes^18,20^. So far, four distinct loci for PM resistance have been identified on four different grape chromosomes in germplasm collected from the Caucasus and China. The presence of strong resistance to powdery mildew in Asian *Vitis* species is at odds with the current theory that the PM originated in North America and was introduced into Europe and the rest of the world in the mid-nineteenth century during a period of frequent trade activity^12,13^. Such a time frame would clearly have been insufficient, in evolutionary terms, for Asian *Vitis* species to develop resistance against the pathogen. Considering the accumulated information on the presence of PM resistance in Asian germplasm, the most plausible explanation is that the fungus was present in Asia for a longer time; possibly from the time period when the continental split separated Asia from America. However, there are no historical records from Central Asia regarding the presence of PM, which only supports the argument that PM was a recent introduction^12,13^.

In both continents, a very large amount of diversity for both host species and resistance loci is observed. North America and China are considered to be the principal centers for diversity for *Vitis* species that are locally adapted to diverse ecogeographic regions and have high amounts of genetic diversity^7,8^. Many of the Chinese *Vitis* species possess resistance to PM^14^, which is difficult to understand if both powdery and downy mildew diseases are North American in origin. It is plausible to suggest that both pathogen and host plants coevolved and developed resistance genes to combat the disease independently on two continents over the period of thousands of years. The presence of PM resistance in the wild progenitor subsp. *sylvestris* from multiple countries also suggests they were exposed to the pathogen for a long time period. Most likely, resistance was inadvertently selected during the later stages of domestication. Comparisons of whole-genome sequencing indicate that wild and cultivated grape samples diverged **∼**22,000 years ago^40^. The purposeful cultivation of grapes and dispersion to different geographic regions must have been focused on visual desirable traits (seedlessness, fruit size and color, bunch size and productivity, and most importantly hermaphroditic flowers). The lack of PM resistance in the majority of cultivated grapes indicates that selection may have not been focused on resistance to the disease. Clonal propagation and long generation time also limited the opportunities for sexual recombination and spread of resistance to a wider range of cultivated varieties.

In conclusion, we genetically mapped PM resistance in the wild progenitor of cultivated grapes, *V. vinifera* subsp. *sylvestris*. The genomic position on chromosome 13 and associated markers are similar to the *Ren1* locus, which was identified in cultivated *V. vinifera* grape. The discovery of new alleles of the SSR markers associated with the PM resistance locus prompted us to expand the search for more *V. vinifera*-based resistance, resulting in the identification of 43 resistant subsp. *sylvestris* accessions that were collected as seed lots or cuttings from five different countries in the center of domestication. Allelic diversity with other SSR markers linked to the resistance locus was detected among the PM-resistant subsp. *sylvestris* accessions. Sequence analysis of two alleles of the SC8–0071-14 marker also found a set of SNPs that were present in the wild progenitor and cultivated forms. These results suggest that PM resistance is prevalent in the subsp. *sylvestris* from the Caucasus and the Pan–Caspian region and may have been present at the time of domestication thousands of years ago. Future work is needed to understand the structure and evolution of the underlying resistance genes.

## Materials and methods

### Plant material and DNA extractions

Three F1 populations designated 14–306, 14–307, and 14–314 were the result of crosses between the PM susceptible pistillate *V. vinifera* subsp. *vinifera* breeding line 08326–61 (selfed Cabernet franc, white berry color), and the resistant *V. vinifera* subsp. *sylvestris* accession DVIT3351.27 (staminate flowers and small cluster size) identified in an earlier study^11^. A total of 189 seedlings from the above crosses are maintained at the Department of Viticulture and Enology, University of California, Davis, California.

Six previously reported PM-resistant accessions (subsp. *vinifera*—Late Vavilov, Karadzhandal, Khalchili, Husseine, Sochal and subsp. *sylvestris*—O34–16) were included as controls to verify previous results^11^. Sixteen other subsp*. sylvestris* accessions collected by H.P. Olmo, as cuttings from Afghanistan and Iran, and 107 subsp. *sylvestris* seedlings from 15 seed lots collected from Armenia, Georgia, and Azerbaijan were included in the study (Table 1, Fig. 2). All these accessions are maintained at the Department of Viticulture and Enology, University of California, Davis, California or the USDA-ARS National Clonal Germplasm Repository, Davis, California.

DNA from each sample was extracted using a modified CTAB procedure which excluded the RNase step^11^. Precipitated DNA was dissolved in 1X TE buffer and stored at −20 °C for further use.

### Disease evaluations

A total of 189 seedling plants from the three breeding populations described above were evaluated for disease resistance. Controlled disease evaluations were performed in an unsprayed shaded greenhouse as described by Pap et al. (2016)^20^. Susceptible (*V. vinifera* ‘Carignan’ and 08326–61), and resistant cultivars (*V. vinifera* ‘Karadzhandal’, *Vitis* hybrids ‘Villard Blanc’ and the *Ren4* breeding selection 08–6053–12) were used as controls in each round of disease evaluations. The seedling replicates and control cultivars were randomized and spaced 10 cm apart. Greenhouse temperature was set to range between 23 and 27 °C, and lights were used to maintain a minimum 12 h day length. The C-isolate^30^ was maintained and multiplied on in vitro grown leaves of the highly susceptible Carignan. On average ~70,000 conidia/ml in 0.1% (v/v) Tween solution were used to spray plants using a Preval Sprayer unit (Nakoma Products, Bridgeville, IL). Two people evaluated the plants for disease symptoms after four weeks of culture using a modified OIV-455 scale^51^: (1) no symptoms, (2) one or two small patches of PM on the entire plant, (3) four to five patches of PM, (4) many leaves with patches of PM, and (5) PM covers entire surface of many leaves on the same plant.

PM infections were evaluated microscopically using in vitro cultured detached leaves for 130 accessions following Pap et al.^20^. Four leaves from the third and fourth position were collected, washed and plated onto 0.8% agar in 100 × 15 mm Petri dishes. A settling tower procedure modified from Reifschneider and Boiteux^52^ was used to ensure the leaves received a uniform inoculation. For all in vitro experiments, two people rated PM growth at 14–15 days post inoculation (dpi) using a dissecting microscope (Leica EZ4 D) with the following scale: (1) no hyphae, (2) one or two conidia with hyphae, (3) several conidia with secondary hyphae and establishment of micro colonies, (4) mycelium on entire leaf surface, limited conidiophore, and (5) mycelia coverage is extensive, reproduction is prolific, clearly visible with the naked eye.

### Marker analysis, genetic linkage map, and QTL analysis

Five SSR markers were used to genotype 189 seedlings from segregating populations, and 130 accessions of both subsp. *vinifera* and *sylvestris*. Polymerase chain reactions of 10 μl volume were carried out with fluorescently-labeled forward primers using the standardized thermocycling profile described in an earlier study^11^. Amplified products were combined depending on the amplicon size and fluorescent labels of the markers. They were then run on an ABI 3500 capillary electrophoresis analyzer with GeneScan-500 Liz Size Standard (Life Technologies, Carlsbad, California, USA). GeneMapper 4.1 software (Applied Biosystem Co., Ltd., USA) was used to determine the allele sizes.

A linkage map was created using JoinMap 4.1^53^. The Kosambi mapping function was used to generate centimorgan (cM) distances^54^. In the interval regression mapping the independence LOD (logarithm of the odds) was set to 5–8 with a one-step interval. QTL analysis was carried out with MapQTL 6.0^55^. Interval mapping (IM) analysis was carried out with a regression algorithm to detect QTLs on the genetic map of DVIT3351.27.

### Genetic diversity measures

The microsatellite tool kit software^56^ was used to calculate standard parameters of genetic variability: expected heterozygosity (He); allele frequencies (AF); and observed heterozygosity (Ho) for five markers for 130 accessions described in Table 1. Principal Coordinate Analysis (PCoA) was carried out with DARwin software (version 5.0.158) to determine the number of groups^57^.

### Sequencing of 141/143 alleles that cosegregate with Ren1 locus

A previous study^11^ developed a set of primers around the region of SSR marker SC8–0071-014 that generated a 625 (bp) amplification product. The same set of primers was used in this study to sequence a subset of 23 accessions that carry allele 141 or 143 at marker SC8–0071-14. The sequence information for allele 141 for two accessions (Karadzhandal and O34-16) was reported in the earlier study^11^; we repeated them in this study to allow comparisons of the results. Amplification of genomic DNA was conducted, and PCR products were cloned using the pGEM^®^-T Easy vector system using standard protocols. Eight positive colonies were selected for each accession. DNA was extracted using the Qiagen plasmid mini kit and amplified with SP6 + T7 to verify transformation. Colonies with the expected insert size were amplified with the SC8–0071-014 primers^21^ and genotyped on a 3500 ABI machine as described above (Applied Biosystems, Foster City, CA). For most accessions, two colonies per sample carrying the 141 or 143 allele were selected for sequencing. A total of 58 samples were sequenced using the T7 universal primer. Vector sequences were removed using the SeqMan program within the DNASTAR Lasergene software V9.0. Two sequences of each accession were compared to verify the sequence and only one copy was used for the final alignment. Alignment of sequences was performed with Geneious V9.0. Sequences of ten accessions reported in a previous study^11^ were also included as references.

## Supplementary information

Supplementary Figure 1

Supplementary Table 1

Supplementary Table 2

Supplementary Table 3

Supplementary Table 4
