# Supplementary figures and images for: Genetic mapping and survey of powdery mildew resistance in the wild Central Asian ancestor of cultivated grapevines in Central Asia
Source: Hortic Res. 2020 Jul 1;7:104. doi: 10.1038/s41438-020-0335-z (PMC7326912; doi:10.1038/s41438-020-0335-z)

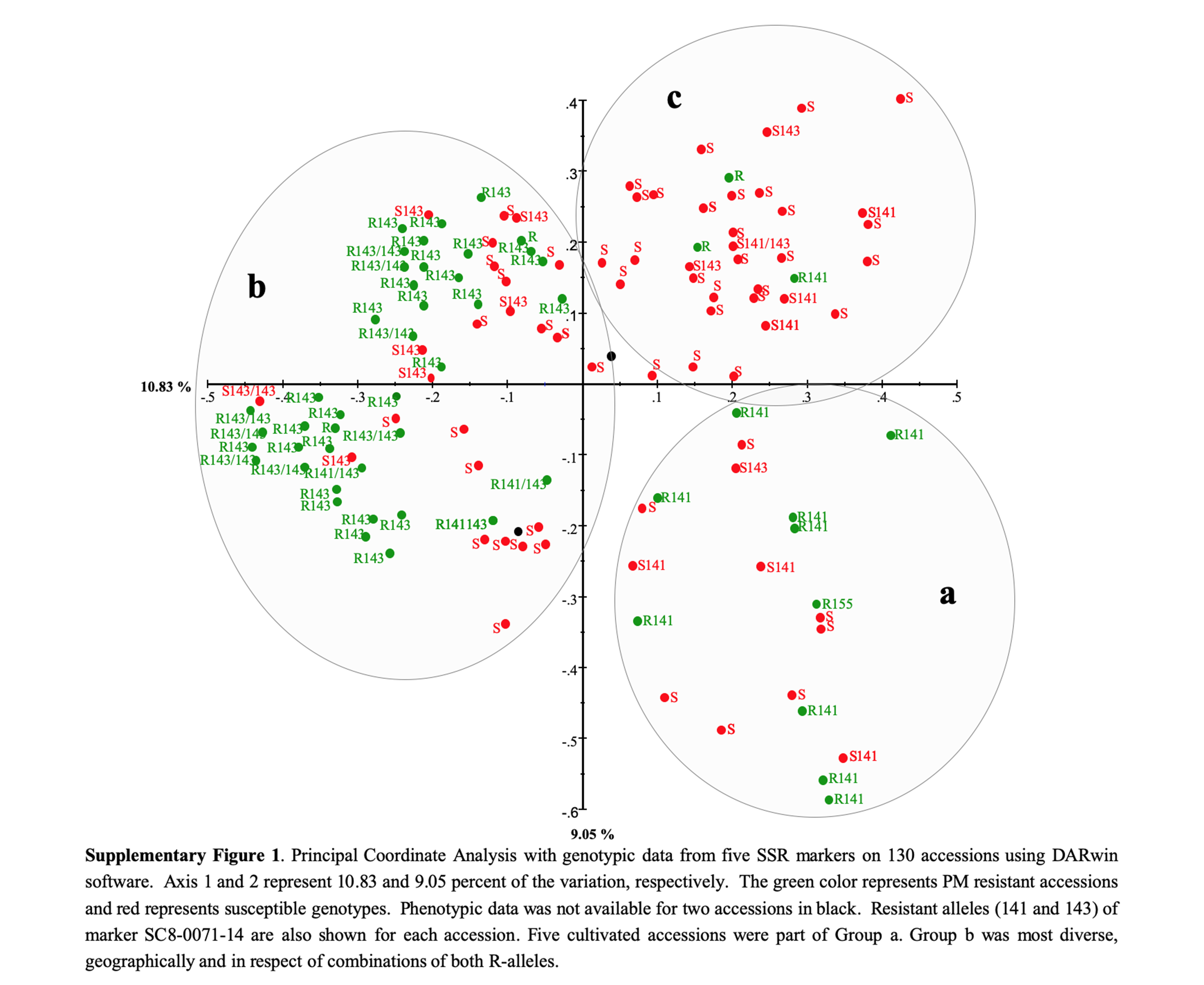

Supplement: Supplementary file 1 — Supplementary Figure 1 [file 41438_2020_335_MOESM1_ESM.png]
